# Enteral versus parenteral nutrition in critically ill patients: an updated systematic review and meta-analysis of randomized controlled trials

**DOI:** 10.1186/s13054-016-1298-1

**Published:** 2016-04-29

**Authors:** Gunnar Elke, Arthur R. H. van Zanten, Margot Lemieux, Michele McCall, Khursheed N. Jeejeebhoy, Matthias Kott, Xuran Jiang, Andrew G. Day, Daren K. Heyland

**Affiliations:** Department of Anaesthesiology and Intensive Care Medicine, University Medical Centre Schleswig-Holstein, Campus Kiel, Arnold-Heller-Str. 3 Haus 12, 24105 Kiel, Germany; Department of Intensive Care, Gelderse Vallei Hospital, Willy Brandtlaan 10, 6716 RP Ede, The Netherlands; Department of Critical Care Medicine, Queen’s University and Clinical Evaluation Research Unit, Kingston General Hospital, Angada 4, K7L 2V7 Kingston, ON Canada; Medical/Surgical ICU, Specialized Complex Care, St Michael’s Hospital, 30 Bond Street, Toronto, ON Canada; Department of Nutritional Sciences, St Michael’s Hospital, 30 Bond Street, Toronto, ON Canada

**Keywords:** Systematic review, Meta-analysis, Nutrition therapy, Enteral nutrition, Parenteral nutrition, Critically ill, Intensive care unit, Infections, Randomized controlled trial

## Abstract

**Background:**

Enteral nutrition (EN) is recommended as the preferred route for early nutrition therapy in critically ill adults over parenteral nutrition (PN). A recent large randomized controlled trial (RCT) showed no outcome differences between the two routes. The objective of this systematic review was to evaluate the effect of the route of nutrition (EN versus PN) on clinical outcomes of critically ill patients.

**Methods:**

An electronic search from 1980 to 2016 was performed identifying relevant RCTs. Individual trial data were abstracted and methodological quality of included trials scored independently by two reviewers. The primary outcome was overall mortality and secondary outcomes included infectious complications, length of stay (LOS) and mechanical ventilation. Subgroup analyses were performed to examine the treatment effect by dissimilar caloric intakes, year of publication and trial methodology. We performed a test of asymmetry to assess for the presence of publication bias.

**Results:**

A total of 18 RCTs studying 3347 patients met inclusion criteria. Median methodological score was 7 (range, 2–12). No effect on overall mortality was found (1.04, 95 % CI 0.82, 1.33, *P* = 0.75, heterogeneity I^2^ = 11 %). EN compared to PN was associated with a significant reduction in infectious complications (RR 0.64, 95 % CI 0.48, 0.87, *P* = 0.004, I^2^ = 47 %). This was more pronounced in the subgroup of RCTs where the PN group received significantly more calories (RR 0.55, 95 % CI 0.37, 0.82, *P* = 0.003, I^2^ = 0 %), while no effect was seen in trials where EN and PN groups had a similar caloric intake (RR 0.94, 95 % CI 0.80, 1.10, *P* = 0.44, I^2^ = 0 %; test for subgroup differences, *P* = 0.003). Year of publication and methodological quality did not influence these findings; however, a publication bias may be present as the test of asymmetry was significant (*P* = 0.003). EN was associated with significant reduction in ICU LOS (weighted mean difference [WMD] -0.80, 95 % CI −1.23, −0.37, *P* = 0.0003, I^2^ = 0 %) while no significant differences in hospital LOS and mechanical ventilation were observed.

**Conclusions:**

In critically ill patients, the use of EN as compared to PN has no effect on overall mortality but decreases infectious complications and ICU LOS. This may be explained by the benefit of reduced macronutrient intake rather than the enteral route itself.

**Electronic supplementary material:**

The online version of this article (doi:10.1186/s13054-016-1298-1) contains supplementary material, which is available to authorized users.

## Background

Artificial nutrition support has evolved into a primary therapeutic intervention to prevent metabolic deterioration and loss of lean body mass with the aim to improve the outcome of critically ill patients. Apart from the timing of initiation and the targeted amount of macronutrients, the route of delivery is viewed as an important determinant of the effect of the nutritional intervention. Using the enteral route is considered to be more physiologic, providing nutritional and various non-nutritional benefits including maintenance of structural and functional gut integrity as well as preserving intestinal microbial diversity [1–3]. The disadvantage of enteral nutrition (EN) is related to a potential lower nutritional adequacy particularly in the acute disease phase and in the presence of gastrointestinal dysfunction [4, 5]. In contrast, parenteral nutrition (PN) may better secure the intended nutritional intake but is associated with more infectious complications, most likely due to hyperalimentation and hyperglycemia, as consistently shown in earlier meta-analyses [6–9]. These clinical data have translated into widespread consensus among current international guideline recommendations [10–13] and expert opinions [14, 15] that the enteral route is preferred in critically ill patients without a contraindication to EN.

Recently, Harvey and coworkers conducted the largest randomized controlled trial (RCT) to date with respect to the effect of the route of nutrition on the outcome of critically ill adult patients [16]. In this pragmatic RCT involving 2388 patients, neither a significant difference in mortality nor infectious complications was found between the patients receiving total PN or EN within 36 hours after admission and up to a maximum of 5 days. These results have challenged the paradigm that EN is superior to PN with regard to clinical outcomes in critical illness.

Therefore, the objective of this study was to perform an updated systematic literature review and meta-analysis of this topic to evaluate the overall effect of the route of nutrition (EN versus PN) on clinical outcomes in adult critically ill patients.

## Methods

### Search strategy and study identification

A literature review was conducted to identify all relevant RCTs published between 1980 and January 2016 in MEDLINE, Embase, CINAHL, the Cochrane Central Register of Controlled Trials, and the Cochrane Database of Systematic Reviews. The following keywords or medical subject headings were used: “randomized”, “clinical trial”, “nutrition support”, “artificial feeding”, “enteral nutrition”, “parenteral nutrition”, “intensive care”, “critical illness”, and “critically ill”. The literature search was not confined to articles written only in English. The authors’ personal files and reference lists of relevant review articles were also reviewed. Neither ethics board approval nor patient consent was required due to the nature of a systematic review.

### Study eligibility criteria

Trials were included only if they met the following characteristics:

1. Type of study: RCT with a parallel group.
2. Population: critically ill adult patients (≥18 years of age), defined as patients admitted to an intensive care unit (ICU). In the event that the information on the study population was unclear, we considered a mortality rate higher than 5 % in the control group to be consistent with critical illness. We excluded RCTs performed in elective surgery patients (such as cardiac surgery patients) even if patients were cared for in an ICU in the postoperative period.
3. Intervention: enteral versus parenteral nutrition
4. Trial outcomes: the trial reported clinically relevant outcomes. Overall mortality was the primary outcome for this meta-analysis. Where available, we extracted data regarding the primary mortality outcome reported as the principal outcome of the study, including ICU, hospital, 28-day mortality or other. Secondary outcomes were infections, ICU and hospital length of stay (LOS), and duration of mechanical ventilation with definitions of infections as defined in the original articles. As in previous meta-analyses conducted by our group, we excluded those trials that reported only nutritional, biochemical, metabolic, or immunologic outcomes.

### Data abstraction

The methodological quality of the included trials was assessed in duplicate by two independent reviewers using a data abstraction form with a scoring system from 0 to 14 according to the following criteria as previously described [8, 17]:

- (a) the extent to which randomization was concealed, (b) blinding, (c) analysis based on the intention-to-treat (ITT) principle, (d) comparability of groups at baseline, (e) extent of follow-up, (f) description of treatment protocol, (g) co-interventions, (h) definition of clinical outcomes.

Disagreement concerning the individual score of each of the defined categories was resolved by consensus between the two reviewers. Moreover, attempts were made to contact the authors of the included trials in order to request further information not contained in the published article, if needed.

### Statistical analysis

Statistical analyses were performed using RevMan 5.3 (Cochrane IMS, Oxford, UK) with a random effects model. All trial data were combined to estimate the pooled risk ratio (RR) with 95 % confidence intervals (CIs) for mortality and infections and overall weighted mean difference (WMD) with 95 % confidence intervals for LOS and mechanical ventilation data. We calculated pooled RRs using the Mantel-Haenszel estimator, and WMDs were estimated by the inverse variance approach. The random effects model of DerSimonian and Laird was applied to estimate variances for the Mantel-Haenszel and inverse variance estimators [18]. In case RRs were undefined they were excluded for studies with no event in either arm. Heterogeneity testing was performed using a weighted Mantel-Haenszel χ^2^ test and quantified by the heterogeneity I^2^ statistic as implemented in RevMan. Differences between subgroups were analyzed using the test of subgroup differences described by Deeks et al. [19], and the results expressed using the *P* values.

Generating funnel plots and testing asymmetry of outcomes using the method proposed by Rücker et al. [20] addressed possible publication bias. We considered *P* < 0.05 to be statistically significant.

### Subgroup analyses

A predefined subgroup analysis was performed to further explore whether the treatment effect of either route is associated with significant differences in the caloric intake across the study groups (EN compared to PN). A priori, we hypothesized that a possible negative treatment effect of PN on mortality, infectious complications and length of stay is related to a higher caloric intake. We used the reported significance level on caloric intake across groups from each study to determine the allocation to the subgroup. We also assessed the effect of trial publication date and trial quality on the outcome based on the hypothesis that older or methodologically weaker studies tended to yield a more negative treatment effect of PN. For this purpose, we designated trials with a publication date later than 1995 (median year 1994–1995) as newer and trials with a methodological score of more than 7 (median score) as methodologically stronger, respectively. In addition, we conducted a sensitivity analysis excluding two studies [21, 22] we were uncertain if the patients were truly critically ill [21] and about the trial results [22] and authors did not respond to attempts to obtain clarification.

## Results

### Study identification and selection

The literature search identified 83 potentially eligible randomized controlled trials, of which 65 were excluded for the following reasons (see Table A1 in Additional file 1): (a) patients not considered to be adult critically ill patients (*N* = 42), (b) no relevant clinical outcomes meeting inclusion criteria reported (*N* = 4), (c) being duplicate studies, reviews of published trials or subgroups of included studies (*N* = 11), (d) non-randomized or pseudo-randomized study design (*N* = 7), and/or (e) control group received a non-standard enteral formula (*N* = 1).

Thus, 18 RCTs with a total number of 3347 critically ill adult patients were finally included in the meta-analysis, whereof 1681 patients were treated with EN and 1666 patients with PN.

The median methodological score of the included 18 RCTs was 7 (range, 2–12) of which 10 RCTs were rated with a score ≤7 and 8 RCTs with a score >7. The median year of publication was 1994–1995 with 10 RCTs published before or in 1995 and 8 RCTs after 1995. All results were based on the individual trial data shown in Tables 1 and 2.

**Table 1:** Included randomized controlled trials of enteral versus parenteral nutrition in critically ill patients

Author	Year	Population	Setting	Total patients^a^	EN group	PN group	Reference
Rapp et al.	1983	Head-injured patients	Single-center	38	18	20	[37]
Adams et al.	1986	Critically ill trauma	Single-center	46	23	23	[38]
Young et al.	1987	Brain-injured patients	Single-center	51	28	23	[39]
Peterson et al.	1988	Critically ill patients with abdominal trauma	Single-center	59	29	30	[40]
Cerra et al.	1988	Critically ill patients	Single-center	70	33	37	[41]
Moore et al.	1989	Abdominal trauma	Single-center	75	39	36	[42]
Kudsk et al.	1992	Abdominal trauma	Single-center	98	52	46	[43]
Dunham et al.	1994	Blunt trauma	Single-center	28^b^	12	16	[44]
Borzotta et al.	1994	Closed head injury	Single-center	59	36	23	[45]
Hadfield et al.	1995	Mixed ICU medical-surgical	Single-center	24	13	11	[46]
Kalfarentzos et al.	1997	Severe acute pancreatitis	Single-center	38	18	20	[47]
Woodcock et al.	2001	ICU patients requiring nutrition support	Single-center	38	17	21	[27]
Casas et al.	2007	Severe acute pancreatitis	Single-center	22	11	11	[48]
Chen et al.	2011	Medical ICU	Single-center	98^b^	49	49	[49]
Justo Meirelles et al.	2011	Traumatic brain injury	Single-center	22	12	10	[21]
Wang et al.	2013	Surgical ICU (severe acute pancreatitis)	Single-center	121^b^	61	60	[22]
Sun et al.	2013	Surgical ICU (severe acute pancreatitis)	Single-center	60	30	30	[50]
Harvey et al.	2014	Mixed medical-surgical	Multi-center	2400	1200	1200	[16]

*EN* enteral nutrition *ICU* intensive care unit *PN* parenteral nutrition

^a^Total number includes number of ICU patients randomized in the trial, even if analysis was not according to intention-to-treat principle

^b^Patients randomized to a third intervention group (combined enteral and parenteral nutrition) of the concerned trial were excluded from this meta-analysis

**Table 2:** Methodology and relevant outcome parameters of the included randomized clinical trials of enteral versus parenteral nutrition in critically ill patients

Study	Methods (score)	Mortality, N (%)^a^		Infections, N (%)^b^		LOS, days, mean ± SD (N)		Mechanical ventilation, days, mean ± SD (N)		Caloric intake^c^	
		EN	PN	EN	PN	EN	PN	EN	PN	EN	PN
1. Rapp et al*.* 1983 [37]	C.Random: not sure	9/18 (50)	3/20 (15)	NR		Hospital 49.4^d^	Hospital 52.6^d^	10.3^d^	10.4^d^	685 *P* = 0.001	1750
	ITT: no										
	Blinding: no (4)										
2. Adams et al. 1986 [38]	C.Random: not sure	1/23 (4)	3/23 (13)	15/23 (65)	17/23 (74)	ICU 13 ± 11 (19)	ICU 10 ± 10 (17)	12 ± 11 (17)	10 ± 10 (13)	2088 NS^f^	2572
	ITT: yes					Hospital 30 ± 21 (19)	Hospital 31 ± 29 (17)				
	Blinding: no (8)										
3. Young et al. 1987 [39]	C.Random: not sure	10/28 (36)	10/23 (43)	5/28 (18)	4/23 (17)	NR		NR		1671 *P* = 0.02	2299
	ITT: no										
	Blinding: no (6)										
4. Peterson et al. 1988 [40]	C.Random: not sure	NR		2/21 (10)	8/25 (32)	ICU 3.7 ± 0.8 (21)	ICU 4.6 ± 1.0 (25)	NR		Kcal on day 5	
										2204 *P* = 0.04	2548
	ITT: no					Hospital 13.2 ± 1.6 (21)	Hospital 14.6 ± 1.9 (24)				
	Blinding: no (5)										
5. Cerra et al. 1988 [41]	C.Random: not sure	ICU 7/31 (22)	ICU 8/35 (23)	NR		NR		NR		Non-protein kcal	
										1684 NS^f^	2000
	ITT: no										
	Blinding: no (2)										
6. Moore et al. 1989 [42]	C.Random: yes	NR		5/29 (17)	11/30 (37)	NR		NR		Non-protein kcal on day 5	
	ITT: no									1847 *P* = 0.01	2261
	Blinding: no (10)										
7. Kudsk et al. 1992 [43]	C.Random: not sure	ICU 1/51 (2)	ICU 1/45 (2)	9/51 (16)	18/45 (40)	Hospital 20.5 ± 19.9 (51)	Hospital 19.6 ± 18.8 (45)	2.8 ± 4.9 (51)	3.2 ± 6.7 (45)	Kcal/kg/d	
	ITT: no									15.7 *P* < 0.05	19.1
	Blinding: single (10)										
8. Dunham et al. 1994 [44]	C.Random: not sure	1/12 (7)	1/15 (8)	NR		NR		NR		NS^f^	
	ITT: no										
	Blinding: no (8)										
9. Borzotta et al. 1994 [45]	C.Random: not sure	5/28 (18)	1/21 (5)	51/28 (28)	39/21 (21)	Hospital^e^ 39 ± 23.1	Hospital^e^ 36.9 ± 14	NR		2097 NS^f^	1961
	ITT: no										
	Blinding: no (6)										
10. Hadfield et al. 1995 [46]	C.Random: not sure	ICU 2/13 (15)	ICU 6/11 (55)	NR		NR		NR		NS^f^	
	ITT: no										
	Blinding: no (7)										
11. Kalfarentzos et al. 1997 [47]	C.Random: not sure	ICU 1/18 (6)	ICU 2/20 (10)	5/18 (28)	0/20 (50)	ICU 11 (5–21)^d^	ICU 12 (5–24)^d^	15 (6–16)^d^	11 (7–31)^d^	Non-protein kcal/kg/d	
	ITT: no					Hospital 40 (25–83)^d^	Hospital 39 (22–73)^d^			24.1 NS^f^	24.5
	Blinding: single (9)										
12. Woodcock et al. 2001 [27]	C.Random: yes	9/17 (53)	5/21 (24)	6/16 (38)	11/21 (52)	33.2 ± 43 (16)	27.3 ± 18.7 (18)	NR		% caloric target (30 kcal/kg/d) achieved	
	ITT: yes									54.1 *P* < 0.001	96.7
	Blinding: single (12)										
13. Casas et al. 2007 [48]	C.Random: no/unsure	Hospital 0/11 (0)	Hospital 2/11 (18)	1/11 (9)	3/11 (27)	Hospital 30.2 (average)	Hospital 30.7 (average)	NR		Kcal/kg/d	
	ITT: Yes									20.1 NS^f^	20.8
	Blinding: no (8)										
14. Chen et al. 2011 [49]	C.Random: yes	20-day 11/49 (22)	20-day 10/49 (20)	5/49 (10)	18/49 (37)	ICU 9.09 ± 2.75	ICU 9.60 ± 3.06	7.95 ± 2.11	8.23 ± 2.42	NR	
	ITT: yes										
	Blinding: no (7)					Hospital 23.32 ± 5.6	Hospital 22.24 ± 3.27				
15. Justo Meirelles et al. 2011 [21]	C.Random: no	Not specified 1/12 (8.3)	Not specified 1/10 (10)	Total infectious complications 2/12 (16.7)	Total infectious complications 4/10 (40)	ICU 14 (5–26)	ICU 14 (6–24)	NR		Cumulative kcal over 5d	
	ITT: no			Pneumonia 2/12 (16.7)	Pneumonia 2/10 (20)					5985 *P* = 0.34	6586
	Blinding: no (5)			Sepsis 0	Sepsis 2/10 (20)						
16. Wang et al. 2013 [22]	C.Random: no	Hospital 3/61 (5)	Hospital 7/60 (12)	Pancreatic sepsis 13/61 (21)	Pancreatic sepsis 24/60 (40)	NR		NR		NR	
	ITT: no										
	Blinding: double (7)			MODS 15/61 (24.6)	MODS 22/60 (36.7)						
17. Sun et al. 2013 [50]	C.Random: no	Hospital 2/30 (7)	Hospital 1/30 (3)	Pancreatic 3/30 (10)	Pancreatic 10/30 (33)	ICU 9 (5–14)	ICU 12 (8–21)	NR		NR	
	ITT: no			MODS 5/30 (17)	MODS 13/30 (43)						
	Blinding: no (6)			SIRS 12/30 (40)	SIRS 22/30 (73)						
18. Harvey et al. 2014 [16]	C.Random: yes	ICU 352/1197 (29.4)	ICU 317/1190 (26.6)	Total infectious complications 194/1197 (16.2)^g^	Total infectious complications 194/1191 (16.3)^g^	ICU 11.3 ± 12.5 (1197)	ICU 12 ± 13.5 (1190)	8.2 ± 9.3 (1197)	8.7 ± 11.5 (1189)	Cumulative kcal/kg/d over 5d	
	ITT: yes	Hospital 450/1186 (37.9)	Hospital 431/1185 (36.4)	Pneumonia 143/1197 (11.9)	Pneumonia 135/1191 (11.3)	Hospital 26.8 ± 33.2 (1186)	Hospital 27.5 ± 33.9 (1185)			74 NS^f^	89
		30-day 409/1195 (34.2)	30-day 393/1188 (33.1)	Bloodstream infections 21/1197 (1.8)	Bloodstream infections 27/1191 (2.9)						
	Blinding: no (8)	90-day 464/1188 (39.1)	90-day 442/1184 (37.3)	Surgical infections 12/1197 (1.0)	Surgical infections 10/1191 (0.8)						

Data are presented as total number and percentage for mortality and infections. Data are presented as mean ± standard deviation with total number of patients per group shown in brackets for LOS and mechanical ventilation

*C.Random* concealed randomization, *d* days, *ITT* intention to treat, *kcal* kilocalories, *LOS* length of stay, *MODS* multiple organ dysfunction syndrome, *N* number, *NR* not reported, *NS* not significant, *SIRS* systemic inflammatory response syndrome

^a^Presumed hospital mortality unless otherwise specified

^b^Refers to the number of patients with infections unless otherwise specified

^c^Caloric intake is presented as the mean daily kcal during the studies’ intervention period or as otherwise specified

^d^Median/mean values, no standard deviation reported hence not included in meta-analysis

^e^Presumed hospital length of stay

^f^No data on caloric intake or *P* value provided, respectively but caloric intake reported to be non-significantly different in the manuscript

^g^Data on ICU patients obtained directly from authors

### Effect of EN versus PN on mortality

**Fig. 1:**
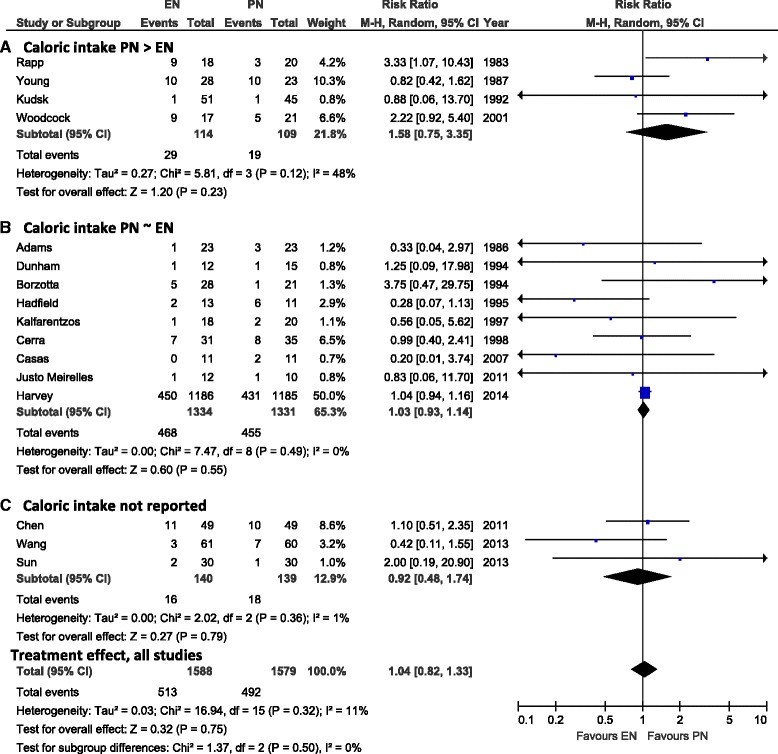
Effects on overall mortality in studies comparing enteral versus parenteral nutrition (*N* = 16 studies). Panel **a** shows the subgroup of aggregated trials in which the caloric intake in the PN group was significantly higher than in the EN group, Panel **b** shows the subgroup of aggregated trials in which the PN and EN groups received similar caloric intake, and Panel **c** includes the trials where caloric intake was not reported. *CI* confidence interval, *EN* enteral nutrition, *M-H* Mantel-Haenszel test, *PN* parenteral nutrition

Aggregating data from all studies reporting on mortality (*N* = 16) there was no difference in overall mortality between the groups receiving EN or PN (RR 1.04, 95 % CI 0.82, 1.33, *P* = 0.75, heterogeneity I^2^ = 11 %) (Fig. 1). In the subgroup analysis where trials were aggregated according to the caloric intake across groups, no effect on mortality was seen in trials (*N* = 4) where the PN group received significantly more calories than the EN group (RR 1.58, 95 % CI 0.75, 3.35, *P* = 0.23, heterogeneity I^2^ = 48 %) or in the nine trials where the caloric intake was reported to be non-significantly different across groups (RR 1.03, 95 % CI 0.93, 1.14, *P* = 0.55, heterogeneity I^2^ = 0 %; test for subgroup differences: *P* = 0.55) (Fig. 1, Panels a and b). Three RCTs did not report the caloric intake across study groups (Fig. 1, Panel c). In the sensitivity analysis excluding two studies [21, 22], there was still no difference in mortality between groups (RR 1.08, 95 % CI 0.83, 1.39, *P* = 0.57, heterogeneity I^2^ = 14 %).

### Effect of EN versus PN on infectious complications

**Fig. 2:**
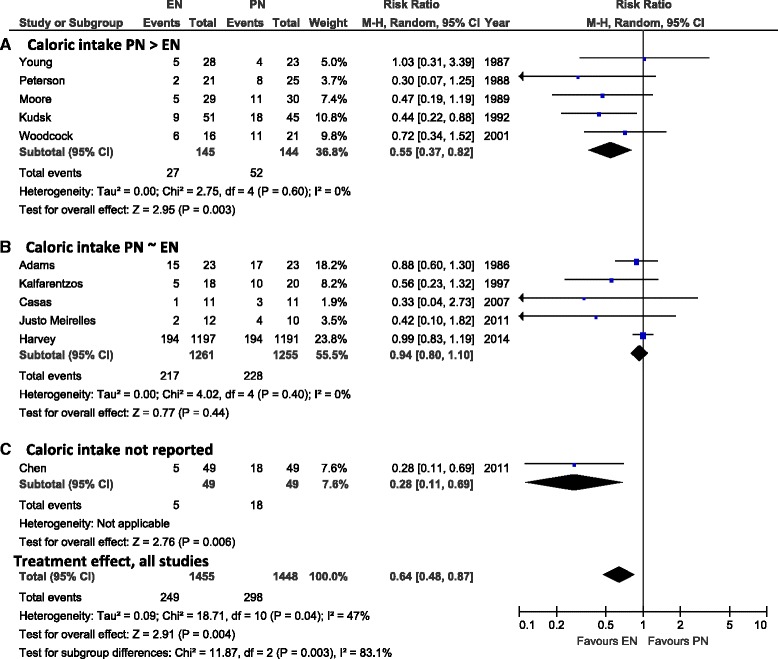
Effects on infectious complications in studies comparing enteral versus parenteral nutrition (*N* = 11 studies). Panel **a** shows the subgroup of aggregated trials in which the caloric intake in the PN group was significantly higher than in the EN group, Panel **b** shows the subgroup of aggregated trials in which the PN and EN groups received similar caloric intake, and Panel **c** includes one trial where caloric intake was not reported. *CI* confidence interval, *EN* enteral nutrition, *M-H* Mantel-Haenszel test, *PN* parenteral nutrition

Eleven trials were aggregated which reported on infectious complications. EN compared to PN was associated with a significant reduction in the incidence of infectious complications (RR 0.64, 95 % CI 0.48, 0.87, *P* = 0.004, heterogeneity I^2^ = 47 %; Fig. 2). The significant difference was also seen in the subgroup analysis of five aggregated trials in which the PN group had a significantly higher caloric intake than the EN group (RR 0.55, 95 % CI 0.37, 0.82, *P* = 0.003, heterogeneity I^2^ = 0 %) but not when the five trials were aggregated where caloric intake was similar between EN and PN groups (RR 0.94, 95 % CI 0.80, 1,10, *P* = 0.44, heterogeneity I^2^ = 0 % [test for subgroup differences: *P* = 0.003]) (Fig. 2, Panels a and b). One trial with data on infectious complications did not report the caloric intake across the EN and PN group (Fig. 2, Panel c). EN compared to PN was still associated with a significant reduction in infectious complications (RR 0.58, 95 % CI 0.41, 0.8, *P* = 0.001, heterogeneity I^2^ = 29 %) in the sensitivity analysis excluding the two studies with inconclusive status of critical illness [21, 22].

### Effect of EN versus PN on ICU and hospital length of stay

**Fig. 3:**
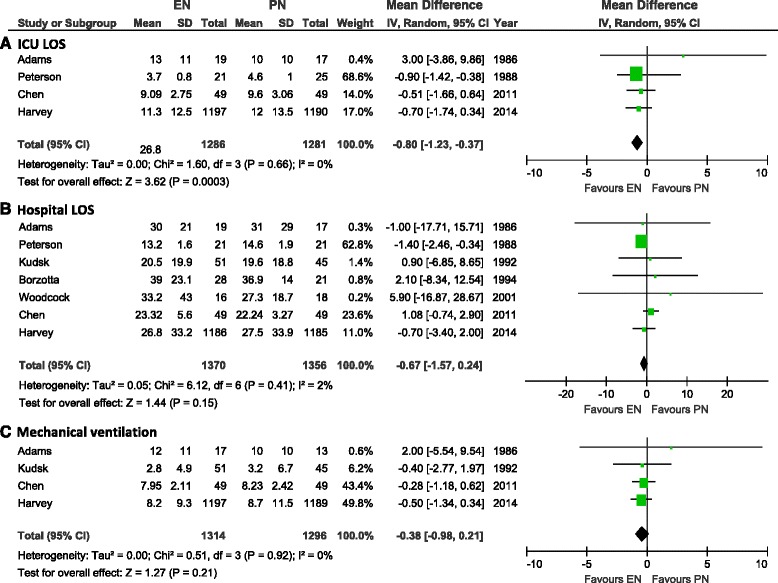
Effects on length of stay and mechanical ventilation in studies comparing enteral versus parenteral nutrition. Panel **a** shows aggregated trials with information on ICU length of stay, Panel **b** shows aggregated trials with information on hospital length of stay. Panel **c** shows aggregated trials with information on length of mechanical ventilation (in mean and standard deviation). *CI* confidence interval, *EN* enteral nutrition, *ICU* intensive care unit, *IV* inverse variance, *LOS* length of stay, *M-H* Mantel-Haenszel test, *PN* parenteral nutrition, *SD* standard deviation

Only four studies reported on ICU LOS (in mean and standard deviation [SD]) and when the data were aggregated, the use of EN was associated with a significant reduction in ICU LOS (WMD −0.80, 95 % CI −1.23, −0.37, *P* = 0.0003, heterogeneity I^2^ = 0 %, Fig. 3, Panel a). In the subgroup analysis aggregating trials according to the caloric intake across groups, the significant difference was not observed in the two trials where caloric intake was similar between EN and PN groups (RR −0.47, 95 % CI −2.23, 1,29, *P* = 0.60, heterogeneity I^2^ = 8 %) (see Figure A1 in Additional file 2). A total of seven RCTs reported on hospital LOS (with mean and standard deviation) where no significant difference was found between EN and PN (WMD −0.67, 95 % CI −1.57, 0.24, *P* = 0.15, heterogeneity I^2^ = 2 %; Fig. 3, Panel b). The non-significant difference remained in the subgroup analysis where trials were aggregated according to the caloric intake across groups (RR −0.67, 95 % CI −1.57, 0.24, *P* = 0.15, heterogeneity I^2^ = 2 %; test for subgroup differences: *P* = 0.08) (see Figure A2 in Additional file 2).

### Effect of EN versus PN on mechanical ventilation

A total of four RCTs reported on length of mechanical ventilation (in mean and standard deviation) with no overall effect observed (WMD −0.38, 95 % CI −0.98, 0.21, *P* = 0.21, heterogeneity I^2^ = 0 %, Fig. 3, Panel c).

### Effect of trial quality and publication date on outcomes and risk of publication bias

**Fig. 4:**
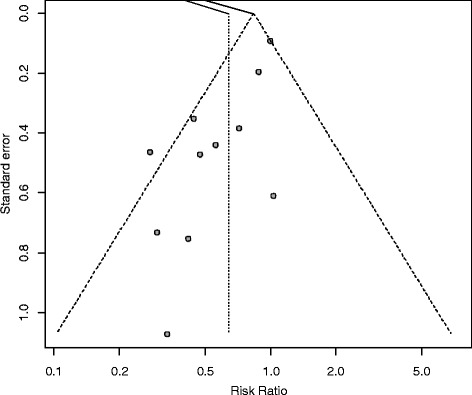
Funnel plot for 11 RCTs reporting the endpoint infectious complications. Test for asymmetry *P* = 0.003

According to the subgroup analyses, there was no effect of either route of nutrition on overall mortality in high-quality trials (*N* = 7 RCTs; RR 1.05, 95 % CI 0.94, 1.16; *P* = 0.38, heterogeneity I^2^ = 0 %) compared to low-quality trials (*N* = 9 RCTs; RR 1.00, 95 % CI 0.62, 1.60; *P* = 1.00, heterogeneity I^2^ = 30 %; test for subgroup differences: *P* = 0.85). This also applied to the comparison of older (*N* = 8 RCTS ≤ 1995; RR 1.01, 95 % CI 0.56, 1.83; *P* = 0.98, heterogeneity I^2^ = 33 %) versus more recent publications (*N* = 8 RCTs > year 1995; RR 1.05; 95 % CI 0.94, 1.16; *P* = 0.38, heterogeneity I^2^ = 0 %; test for subgroup differences: *P* = 0.90). With respect to infectious complications, the positive treatment effect of EN remained significantly independent of methodological trial quality (*N* = 4 RCTs with score ≤ 7; RR 0.42; 95 % CI 0.23, 0.77; *P* = 0.005; heterogeneity I^2^ = 6 % and *N* = 7 RCTs with score > 7; RR 0.76; 95 % CI 0.58, 0.99; *P* = 0.04; heterogeneity I^2^ = 37 %; test for subgroup differences: *P* = 0.08) (see Figure A3 in Additional file 3) and independent of the trial publication date (*N* = 6 RCTs > 1995; RR 0.61; 95 % CI 0.37, 0.99; *P* = 0.05, heterogeneity I^2^ = 55 % and *N* = 5 RCTs ≤ 1995; RR 0.62; 95 % CI 0.39, 0.98; *P* = 0.04, heterogeneity I^2^ = 39 %; test for subgroup differences: *P* = 0.94) (see Figure A4 in Additional file 4). Results regarding the treatment effect of EN versus PN on ICU and hospital LOS in both subgroup analyses also remained concordant compared with the primary analyses. Funnel plots for all outcome measures were created to further test for potential publication bias. The test for asymmetry was found to be significant for the reported endpoint infectious complications (*P* = 0.003) (Fig. 4). No significant differences were found with respect to the remaining endpoints of overall mortality (*P* = 0.61), ICU LOS (*P* = 0.34), hospital LOS (*P* = 0.30) or mechanical ventilation (*P* = 0.65) (data not shown).

## Discussion

This updated meta-analysis on the effect of the route of nutrition (EN versus PN) on clinical outcomes included 18 randomized controlled trials with a total of 3347 randomized critically ill adult patients. Overall, there was no difference in mortality between the two routes of nutrition. EN as compared to PN led to a significant reduction in the number of infectious complications and ICU LOS while no significant effect was found with respect to hospital LOS and mechanical ventilation. However, the positive treatment effect of EN on infectious morbidity and ICU LOS may be attributed to differences in caloric intake between study groups. Furthermore, funnel plot analysis revealed evidence for significant publication bias for the trials reporting on infectious complications.

### Comparison to other meta-analyses

Six previous meta-analyses comparing the effect of EN versus PN on clinical outcomes of critically ill adults reported no significant overall difference in mortality between the two routes of nutrition [6, 7, 9, 23–25]. The meta-analysis by Simpson and Doig [25] including 11 RCTs only showed a mortality benefit of PN in a predefined subgroup analysis where PN was compared to delayed EN (odds ratio [OR] 0.29, 95 % CI 0.12, 0.70, *P* = 0.006; heterogeneity I^2^ = 0 %, statistical heterogeneity *P* = 0.60). In the absence of an overall mortality effect, all of these meta-analyses showed significant reductions in the rate of infectious complications with the use of EN and one meta-analysis also showing a significant reduction of hospital LOS (WMD = 1.20 days; 95 % CI 0.38, 2.03; *P* = 0.004) [9].

In contrast to these previous meta-analyses, our updated results include the data of the recent multicenter RCT (“Calories trial”) by Harvey and coworkers [16]. In this pragmatic trial including 2400 critically ill patients, the use of total PN was compared with EN for a duration of 5 days after ICU admission. The calorie and protein intake was similar, albeit low with respect to the predefined nutrition target in both groups, while initiation of oral feeding was allowed if clinically indicated during the intervention period. Neither were there significant differences in the 90-day mortality primary endpoint nor in the rate of infectious complications or other secondary outcome measures including LOS variables and duration of mechanical ventilation. Why the increased infectious morbidity seen with PN in the former trials and meta-analyses was not observed in this latest largest RCT may have been related to the equally hypocaloric delivery of macronutrients via both routes.

### Effect of dissimilar caloric intake

In contrast to the results of the Calories trial [16], the overall significant positive treatment effect of EN on infectious morbidity and ICU LOS remained in our updated meta-analysis. On the one hand, this overall treatment effect may reflect the positive non-nutritional effects of EN in terms of preservation of gut integrity and intestinal microbial diversity as well as promotion of gut-mediated immunity, all of which may support the systemic immune response [2, 26]. Presumably, the duration of the intervention (5 days) in the Calories trial [16] was likely too short or not intense enough in a population with a mortality rate of 34 % that the beneficial non-nutritional, trophic effects of EN became evident with respect to infectious morbidity and ICU LOS. On the other hand, our subgroup findings support the hypothesis that not the route per se but rather likely the dissimilar amount of calories delivered may have influenced the treatment effect on infectious complications. While there was a significant treatment effect difference in the subgroup of five RCTs where caloric intake was reported to be significantly higher in the PN group, no treatment effect on infectious complications was observed in the five trials with similar caloric intake across the study groups. This latter subgroup observation was largely driven by the Calories trial contributing 23.8 % of the overall estimate [16]. Furthermore, studies showing differences in infectious complications were associated with publication bias. Except for one RCT [27], the caloric intake in the PN group was gradually increased to target in all aggregated trials. Still, PN as compared to EN poses a higher risk of providing macronutrients in excess of the metabolic capacity, particularly in the early phase of illness and in the absence of appropriate metabolic control [28]. Caloric overfeeding per se is regarded to negatively influence outcomes with an increased risk of infectious complications while a restricted delivery of macronutrients or the avoidance of overfeeding may preserve autophagy and thus likely positively influence outcome [29, 30]. Unfortunately, owing to the limited number of trials in which the relation of nutritional intakes and predefined targets were reported, we were neither able to further explore the treatment effect of nutritional adequacy nor hyperglycemia on infectious complications in more detail. With respect to the effect of EN-specific complications such as vomiting, aspiration or diarrhea on clinical outcomes, we were unable to complete another subgroup analysis due to inconsistent reporting in the included RCTs. The Calories trial revealed that PN as compared to EN was associated with a lower rate of vomiting and diarrhea but a higher rate of constipation [16]. However, the impact of EN-specific complications remains inconclusive given the non-significant differences in major clinical outcomes in the Calories trial and two other large RCTs comparing the effect of different EN feeding strategies [31, 32].

### Effect of trial quality and publication bias

Most of the RCTs that were aggregated in our meta-analysis were single-center trials reporting small total number of patients, and ten RCTs were published more than 20 years ago. Based on the hypothesis that older or methodologically weaker studies per se tended to yield the more negative treatment effect of PN, we performed additional subgroup analyses as well as funnel plots to assess risk of publication bias. In the early meta-analysis by Braunschweig et al., the positive treatment effect of EN compared to PN on infection rates was independent of the publication date and trial quality score [6]. While the positive treatment effect of EN on infectious morbidity accordingly appeared to be independent of the publication date and methodological trial quality in our meta-analysis, the funnel plot analysis revealed significant asymmetry with respect to infectious complication rates. This bias is apparently driven by the published smaller trials showing a larger treatment effect weakening the strength of the inference we can make about the overall effect of the route of nutrition on infectious complication rates.

### Strength and limitations

The strengths of our meta-analysis are the comprehensive and most up-to-date search of the worldwide literature without restriction to only English-written articles, the inclusion of data from the largest and most recent RCT by Harvey and coworkers [16], the duplicate data abstraction and specific criteria for searching and analysis, and the analysis of trial quality, publication year, and publication bias. Limitations of our meta-analysis include the missing outcome data points in some of the included trials, and the small number of aggregated trials with data on the clinical endpoints ICU LOS and duration of mechanical ventilation. Further limitations are the variation in reporting the caloric intake, the time of nutrition intervention, and the definitions used for infections with or without adjudication across the trials included in the subgroup analysis. We were also unable to separate the effect of protein intake via both routes on the reported clinical endpoints among the included trials. Thus, there may be other covariates driving the observed findings that were not adjusted for in our meta-analysis. This may also pertain to possible treatment effect differences of EN versus PN among specific subpopulations of critically ill patients, such as those with a different nutritional risk upon ICU admission. The results of our meta-analysis may not be applicable to patients with a relative short-term or absolute contraindication for EN where the treatment effect of PN (as compared to standard care or no nutrition) on clinical outcomes may differ, as shown in two recent large RCTs [33, 34]. Moreover, the effect on long-term functional outcomes were not studied in the aggregated RCTs but are currently viewed as the more appropriate endpoints to be influenced by different nutritional interventions including the route and amount of nutrition [14, 35]. Lastly, we did not examine cost-effectiveness of the two strategies of nutrition due to the inconsistency of reported data in the trials. It is likely that EN will incur less cost as compared to PN as shown by a secondary analysis of the Calories trial [36].

## Conclusions

In this comprehensive and most up-to-date systematic review we found that the route of nutrition (EN versus PN) does not impact mortality in a heterogeneous population of critically ill adult patients. Overall, the use of EN as compared to PN significantly reduced the rate of infectious complications and length of ICU stay. However, the different treatment effect concerning infectious morbidity favouring EN must be interpreted in light of the observed differences in caloric intake across study groups and publication bias of included trials. Although these observations reduce the strength of inference with respect to the negative effects of PN on infectious morbidity, the observed favourable effects of EN on ICU LOS, the ease of access, and lower costs in patients who tolerate EN should be considered. Therefore, in accordance with the most recent guideline recommendations [11, 12], we posit that EN still should be considered the first-line nutritional therapy in adult critically ill patients with a functioning gastrointestinal tract.

## Key messages

- This updated meta-analysis on effects of EN versus PN on clinical outcomes included 18 RCTs with 3347 randomized critically ill patients
- There was no significant difference in mortality between patients fed via EN or PN
- Compared to PN, the use of EN was associated with a significant reduction of infectious complications and ICU LOS
- This positive treatment effect of EN compared to PN may be attributed to differences in caloric intake and significant publication bias among aggregated trials
- EN still should be considered first-line nutritional therapy over PN in critically ill patients with a functioning gastrointestinal tract

## Additional files

Additional file 1: Table A1. Excluded studies of enteral versus parenteral nutrition. (PDF 109 kb) Additional file 2: Figure A1. Subgroup analysis comparing the effect of enteral versus parenteral nutrition according to the caloric intake on length of intensive care unit stay (*N* = 4 studies). Panel A shows the subgroup of aggregated trials in which the caloric intake in the PN group was significantly higher than in the EN group, Panel B shows the subgroup of aggregated trials in which the PN and EN groups received similar caloric intake and Panel C including one trial where caloric intake was not reported. *CI* confidence interval, *EN* enteral nutrition, *M-H* Mantel-Haenszel test, *PN* parenteral nutrition. **Figure A2.** Subgroup analysis comparing the effect of enteral versus parenteral nutrition on length of hospital stay according to the caloric intake (*N* = 7 studies). Panel A shows the subgroup of aggregated trials in which the caloric intake in the PN group was significantly higher than in the EN group, Panel B shows the subgroup of aggregated trials in which the PN and EN groups received similar caloric intake and Panel C including one trial where caloric intake was not reported. *CI* confidence interval, *EN* enteral nutrition, *IV* inverse variance, *M-H* Mantel-Haenszel test, *PN* parenteral nutrition. (PDF 148 kb) Additional file 3: Figure A3. Subgroup analysis comparing the effect of enteral versus parenteral nutrition on infectious complications in higher versus lower quality trials (with the median methodological score 7 as cutoff). *CI* confidence interval, *EN* enteral nutrition, *M-H* Mantel-Haenszel test, *PN* parenteral nutrition. (PDF 87 kb) Additional file 4: Figure A4. Subgroup analysis comparing the effect of enteral versus parenteral nutrition on infectious complications in newer versus older trials (with the publication date 1995 as cutoff). *CI* confidence interval, *EN* enteral nutrition, *M-H* Mantel-Haenszel test, *PN* parenteral nutrition. (PDF 88 kb)

## Abbreviations

C.Random: concealed randomization
CI: confidence interval
EN: enteral nutrition
ICU: intensive care unit
ITT: intention-to-treat
IV: inverse variance
kcal: kilocalories
LOS: length of stay
MODS: multiple organ dysfunction syndrome
N: number
NR: not reported
NS: not significant
OR: odds ratio
PN: parenteral nutrition
RCT: randomized controlled trial
RR: relative risk
SD: standard deviation
SIRS: systemic inflammatory response syndrome
WMD: weighted mean difference
