# Supplementary material for: Enteral versus parenteral nutrition in critically ill patients: an updated systematic review and meta-analysis of randomized controlled trials
Source: Crit Care. 2016 Apr 29;20:117. doi: 10.1186/s13054-016-1298-1 (PMC4851818; doi:10.1186/s13054-016-1298-1)
Supplement: Additional file 1: Table A1. — Excluded studies of enteral versus parenteral nutrition. (PDF 109 kb) [file 13054_2016_1298_MOESM1_ESM.pdf]

Enteral versus parenteral nutrition in critically ill patients: an updated systematic review and meta-analysis of randomized controlled trials

Gunnar Elke, Arthur R.H. van Zanten, Margot Lemieux, Michele McCall, Khursheed N. Jeejeebhoy, Matthias Kott, Xuran Jiang, Andrew G. Day, Daren K. Heyland

Additional file 1

Table A1. Excluded studies of enteral versus parenteral nutrition

Number	Author	Year	Reasons for exclusion	Reference
1	Lim et al	1981	Patients not considered to be adult critically ill patients	[1]
2	Sako et al.	1981	Patients not considered to be adult critically ill patients	[2]
3	Bauer et al.	1984	Patients not considered to be adult critically ill patients	[3]
4	Quayle et al.	1984	Patients not considered to be adult critically ill patients	[4]
5	Seri et al.	1984	Patients not considered to be adult critically ill patients	[5]
6	Wiedeck et al.	1984	Patients not considered to be adult critically ill patients	[6]
7	Costalat et al.	1985	Patients not considered to be adult critically ill patients	[7]
8	Bower et al.	1986	Patients not considered to be adult critically ill patients	[8]
9	Fletcher et al.	1986	Patients not considered to be adult critically ill patients	[9]
10	Hadley et al.	1986	Pseudo-randomized study design	[10]
11	Young et al.	1987	No relevant clinical outcomes meeting inclusion criteria reported	[11]
12	Greenberg et al.	1988	Patients not considered to be adult critically ill patients	[12]
13	Hamaoui et al.	1990	Patients not considered to be adult critically ill patients	[13]
14	Moore et al.	1992	Systematic review, individual studies were reviewed for eligibility	[14]
15	Von Meyenfeldt et al.	1992	Patients not considered to be adult critically ill patients	[15]
16	Gonzalez-Huix et al.	1993	Patients not considered to be adult critically ill patients	[16]
17	Iovinelli et al.	1993	Patients not considered to be adult critically ill patients	[17]
18	Kudsk KA	1994	Duplicate of Kudsk et al. 1992 [Reference 43 in manuscript]	[18]
19	Wicks et al.	1994	Patients not considered to be adult critically ill patients	[19]
20	Baigrie et al.	1996	Patients not considered to be adult critically ill patients	[20]
21	Hernandez-Aranda et al.	1996	Patients not considered to be adult critically ill patients	[21]
22	Suchner et al.	1996	No relevant clinical outcomes meeting inclusion criteria reported	[22]
23	Georgiannos et al.	1997	Patients not considered to be adult critically ill patients	[23]
24	Gioanotti et al.	1997	Patients not considered to be adult critically ill patients	[24]

25	McClave et al.	1997	Patients not considered to be adult critically ill patients	[25]
26	Reynolds et al.	1997	Patients not considered to be adult critically ill patients	[26]
27	Sand et al.	1997	Patients not considered to be adult critically ill patients	[27]
28	Shirabe et al.	1997	Patients not considered to be adult critically ill patients	[28]
29	Braga et al.	1998	Patients not considered to be adult critically ill patients	[29]
30	Windsor et al.	1998	Patients not considered to be adult critically ill patients	[30]
31	Oláh et al.	2000	Patients not considered to be adult critically ill patients	[31]
32	Bozetti et al.	2001	Patients not considered to be adult critically ill patients	[32]
33	Braga et al.	2001	Patients not considered to be adult critically ill patients	[33]
34	Braunschweig et al.	2001	Systematic review, individual studies were reviewed for eligibility	[34]
35	Heyland et al.	2001	Patients not considered to be adult critically ill patients	[35]
36	Pacelli et al.	2001	Patients not considered to be adult critically ill patients	[36]
37	Abou-Assi et al.	2002	Patients not considered to be adult critically ill patients	[37]
38	Huang et al.	2000	Non-randomized study design	[38]
39	Oláh et al.	2002	Pseudo-randomized study design	[39]
40	Rayes et al.	2002	Patients not considered to be adult critically ill patients	[40]
41	Gupta et al.	2003	Patients not considered to be adult critically ill patients	[41]
42	Zhao et al.	2003	No relevant clinical outcomes meeting inclusion criteria reported	[42]
43	Louie et al.	2005	Patients not considered to be adult critically ill patients	[43]
44	Peter et al.	2005	Systematic review, individual studies were reviewed for eligibility	[44]
45	Simpson et al.	2005	Systematic review, individual studies were reviewed for eligibility	[45]
46	Eckerwall et al.	2006	Patients not considered to be adult critically ill patients	[46]
47	Petrov et al.	2006	Patients not considered to be adult critically ill patients	[47]
48	Radrizzani et al.	2006	Control group received a non standard enteral formula	[48]
49	Chen et al.	2007	No relevant clinical outcomes meeting inclusion criteria reported	[49]
50	Petrov et al.	2007	Systematic review, individual studies were reviewed for eligibility	[50]
51	Tian et al.	2007	Patients not considered to be adult critically ill patients	[51]
52	Cao et al.	2008	Systematic review, individual studies were reviewed for eligibility	[52]
53	Farimani and Bajestani	2008	Patients not considered to be adult critically ill patients	[53]
54	Lam et al.	2008	Pseudo-randomized study design	[54]

55	Petrov et al.	2008	Systematic review, individual studies were reviewed for eligibility	[55]
56	Cheng et al.	2009	Patients not considered to be adult critically ill patients	[56]
57	Doley et al.	2009	Pseudo-randomized study design	[57]
58	Nagata et al.	2009	Patients not considered to be adult critically ill patients	[58]
59	Petrov et al.	2009	Systematic review, individual studies were reviewed for eligibility	[59]
60	Ryu et al.	2009	Patients not considered to be adult critically ill patients	[60]
61	Vieira et al.	2010	Non-randomized study design	[61]
62	Altintas et al.	2011	Pseudo-randomized study design	[62]
63	Cangelosi et al.	2011	Systematic review, individual studies were reviewed for eligibility	[63]
64	Klek et al.	2011	Patients not considered to be adult critically ill patients	[64]
65	Wang et al.	2013	Systematic review, individual studies were reviewed for eligibility	[65]

References

1. Lim ST, Choa RG, Lam KH, et al. Total parenteral nutrition versus gastrostomy in the preoperative preparation of patients with carcinoma of the oesophagus. Br J Surg 1981; 68:69-72.
2. Sako K, Loré JM, Kaufman S, et al. Parenteral hyperalimentation in surgical patients with head and neck cancer: a randomized study. J Surg Oncol 1981; 16:391-402.
3. Bauer E, Graber R, Brodike R, et al. Ernahrungsphysiologische, immunologische und klinische parameter bei prospektiv randomisierten patienten unter enteraler oder parenteraler ernahrungstherapie nach dickdarmoperationen. Infusionstherapie 1984; 11:165-167.
4. Quayle AR, Mangnall DC, RG. A comparison of immediate post-operative enteral and parenteral nutrition in patients with gastric carcinoma. Clin Nutr 1984; 3:35-39.
5. Seri S, Aquilio E. Effects of early nutritional support in patients with abdominal trauma. Ital J Surg Sci 1984; 14:223-227.
6. Wiedeck H, Merkle N, Herfarth C, Grünert A. Postoperative enteral feeding following resection of the colon. Anaesthesist 1984; 33:63-67.
7. Costalat G, Vernhet J. Early postoperative enteral nutrition using a jejunal catheter in major digestive surgery. Comparison with total parenteral nutrition. Chirurgie 1985; 111:708-714.
8. Bower RH, Talamini MA, Sax HC, et al. Postoperative enteral vs parenteral nutrition. A randomized controlled trial. Arch Surg 1986; 121:1040-1045.
9. Fletcher JP, Little JM. A comparison of parenteral nutrition and early postoperative enteral feeding on the nitrogen balance after major surgery. Surgery 1986; 100:21-24.
10. Hadley MN, Grahm TW, Harrington T, et al. Nutritional support and neurotrauma: a critical review of early nutrition in forty-five acute head injury patients. Neurosurgery 1986; 19:367-373.
11. Young B, Ott L, Haack D et al. Effect of total parenteral nutrition upon intracranial pressure in severe head injury. J Neurosurg 1987; 67:76-80.
12. Greenberg GR, Fleming CR, Jeejeebhoy KN, et al. Controlled trial of bowel rest and nutritional support in the management of Crohn's disease. Gut 1988; 29:1309-1315.
13. Hamaoui E, Lefkowitz R, Olender L et al. Enteral nutrition in the early postoperative period: a new semi-elemental formula versus total parenteral nutrition. J Parenter Enteral Nutr 1990; 14:501- 507.
14. Moore FA, Feliciano DV, Andrassy RJ et al. Early enteral feeding, compared with parenteral, reduces postoperative septic complications. The results of a meta-analysis. Ann Surg 1992; 216:172-183.
15. Von Meyenfeldt MF, Meijerink WJ, Rouflart MM, et al. Perioperative nutritional support: a randomised clinical trial. Clin Nutr 1992; 11:180-186.
16. González-Huix F, Fernández-Bañares F, Esteve-Comas M et al. Enteral versus parenteral nutrition as adjunct therapy in acute ulcerative colitis. Am J Gastroenterol 1993; 88:227-232.

17. Iovinelli G, Marsili I, Varrassi G. Nutrition support after total laryngectomy. J Parenter Enteral Nutr 1993; 17:445-448.
18. Kudsk KA. Gut mucosal nutritional support--enteral nutrition as primary therapy after multiple system trauma. Gut 1994; 35:S52-4.
19. Wicks C, Somasundaram S, Bjarnason I, et al. Comparison of enteral feeding and total parenteral nutrition after liver transplantation. Lancet 1994; 344:837-840.
20. Baigrie RJ, Devitt PG, Watkin DS. Enteral versus parenteral nutrition after oesophagogastric surgery: a prospective randomized comparison. Aust N Z J Surg 1996; 66:668-670.
21. Hernández-Aranda JC, Gallo-Chico B, Ramírez-Barba EJ. Nutritional support in severe acute pancreatitis. Controlled clinical trial. Nutr Hosp 1996; 11:160-166.
22. Suchner U, Senftleben U, Eckart T, et al. Enteral versus parenteral nutrition: effects on gastrointestinal function and metabolism. Nutrition 1996; 12:13-22.
23. Georgiannos SN, Renaut AJ, Goode AW. Short-term restorative nutrition in malnourished patients: pro's and con's of intravenous and enteral alimentation using compositionally matched nutrients. Int Surg 1997; 82:301-306.
24. Gianotti L, Braga M, Vignali A, et al. Effect of route of delivery and formulation of postoperative nutritional support in patients undergoing major operations for malignant neoplasms. Arch Surg 1997; 132:1222-9; discussion 1229.
25. McClave SA, Greene LM, Snider HL, et al. Comparison of the safety of early enteral vs parenteral nutrition in mild acute pancreatitis. J Parenter Enteral Nutr 1997; 21:14-20.
26. Reynolds JV, Kanwar S, Welsh FK, et al. 1997 Harry M. Vars Research Award. Does the route of feeding modify gut barrier function and clinical outcome in patients after major upper gastrointestinal surgery. J Parenter Enteral Nutr 1997; 21:196-201.
27. Sand J, Luostarinen M, Matikainen M. Enteral or parenteral feeding after total gastrectomy: prospective randomised pilot study. Eur J Surg 1997; 163:761-766.
28. Shirabe K, Matsumata T, Shimada M, et al. A comparison of parenteral hyperalimentation and early enteral feeding regarding systemic immunity after major hepatic resection--the results of a r andomized prospective study. Hepatogastroenterology 1997; 44:205-209.
29. Braga M, Gianotti L, Vignali A, et al. Artificial nutrition after major abdominal surgery: impact of route of administration and composition of the diet. Crit Care Med 1998; 26:24-30.
30. Windsor AC, Kanwar S, Li AG, et al. Compared with parenteral nutrition, enteral feeding attenuates the acute phase response and improves disease severity in acute pancreatitis. Gut 1998; 42:431-435.
31. Oláh A, Pardavi G, Belágyi T. [Early jejunal feeding in acute pancreatitis: prevention of septic complications and multiorgan failure]. Magy Seb 2000; 53:7-12.
32. Bozzetti F, Braga M, Gianotti L, et al. Postoperative enteral versus parenteral nutrition in malnourished patients with gastrointestinal cancer: a randomised multicentre trial. Lancet 2001; 358:1487-1492.
33. Braga M, Gianotti L, Gentilini O, et al. Early postoperative enteral nutrition improves gut oxygenation and reduces costs compared with total parenteral nutrition. Crit Care Med 2001; 29:242-248.
34. Braunschweig CL, Levy P, Sheean PM, Wang X. Enteral compared with parenteral nutrition: a meta-analysis. Am J Clin Nutr 2001; 74:534-542.
35. Heyland DK, Montalvo M, MacDonald S, et al. Total parenteral nutrition in the surgical patient: a meta-analysis. Can J Surg 2001; 44:102-111.
36. Pacelli F, Bossola M, Papa V, et al. Enteral vs parenteral nutrition after major abdominal surgery: an even match. Arch Surg 2001; 136:933-936.
37. Abou-Assi S, Craig K, O'Keefe SJ. Hypocaloric jejunal feeding is better than total parenteral nutrition in acute pancreatitis: results of a randomized comparative study. Am J Gastroenterol 2002; 97:2255-2262.
38. Huang YC, Yen CE, Cheng CH, Jih KS, Kan MN. Nutritional status of mechanically ventilated critically ill patients: comparison of different types of nutritional support. Clin Nutr 2000; 19:101-107.
39. Oláh A, Pardavi G, Belágyi T, et al. Early nasojejunal feeding in acute pancreatitis is associated with a lower complication rate. Nutrition 2002; 18:259-262.
40. Rayes N, Hansen S, Seehofer D, et al. Early enteral supply of fiber and Lactobacilli versus conventional nutrition: a controlled trial in patients with major abdominal surgery. Nutrition 2002; 18:609-615.
41. Gupta R, Patel K, Calder PC, et al. A randomised clinical trial to assess the effect of total enteral and total parenteral nutritional support on metabolic, inflammatory and oxidative markers in patients with predicted severe acute pancreatitis (APACHE II > or =6). Pancreatology 2003; 3:406-413.
42. Zhao G, Wang CY, Wang F, Xiong JX. Clinical study on nutrition support in patients with severe acute pancreatitis. World J Gastroenterol 2003; 9:2105-2108.

43. Louie BE, Noseworthy T, Hailey D, et al. 2004 MacLean-Mueller prize enteral or parenteral nutrition for severe pancreatitis: a randomized controlled trial and health technology assessment. Can J Surg 2005; 48:298-306.
44. Peter JV, Moran JL, Phillips-Hughes J. A metaanalysis of treatment outcomes of early enteral versus early parenteral nutrition in hospitalized patients. Crit Care Med 2005; 33:213-20; discussion 260.
45. Simpson F, Doig GS. Parenteral vs. enteral nutrition in the critically ill patient: a meta-analysis of trials using the intention to treat principle. Intensive Care Med 2005; 31:12-23.
46. Eckerwall GE, Axelsson JB, Andersson RG. Early nasogastric feeding in predicted severe acute pancreatitis: A clinical, randomized study. Ann Surg 2006; 244:959-65.
47. Petrov MS, Kukosh MV, Emelyanov NV. A randomized controlled trial of enteral versus parenteral feeding in patients with predicted severe acute pancreatitis shows a significant reduction in mortality and in infected pancreatic complications with total enteral nutrition. Dig Surg 2006; 23:336-44.
48. Radrizzani D, Bertolini G, Facchini R, et al. Early enteral immunonutrition vs. parenteral nutrition in critically ill patients without severe sepsis: a randomized clinical trial. Intensive Care Med 2006; 32:1191-1198.
49. Chen Z, Wang S, Yu B, Li A. A comparison study between early enteral nutrition and parenteral nutrition in severe burn patients. Burns 2007; 33:708-712.
50. Petrov MS, Zagainov VE. Influence of enteral versus parenteral nutrition on blood glucose control in acute pancreatitis: a systematic review. Clin Nutr 2007; 26:514-523.
51. Tian BL, Cao HF, Hu WM, et al. The morphological alterations of jejunal mucosa accepting early enteral nutrition for post-operative patients with severe acute pancreatitis. Sichuan Da Xue Xue Bao Yi Xue Ban 2007; 38:264-267.
52. Cao Y, Xu Y, Lu T, Gao F, Mo Z. Meta-analysis of enteral nutrition versus total parenteral nutrition in patients with severe acute pancreatitis. Ann Nutr Metab 2008; 53:268-275.
53. Farimani MRV, Bajestani NN. Comparison of early enteral feeding versus parenteral nutrition after resection of esophageal cancer. J Crit Care 2008; 23:448.
54. Lam NN, Tien NG, Khoa CM. Early enteral feeding for burned patients--an effective method which should be encouraged in developing countries. Burns 2008; 34:192-196.
55. Petrov MS, Pylypchuk RD, Emelyanov NV. Systematic review: nutritional support in acute pancreatitis. Aliment Pharmacol Ther 2008; 28:704-712.
56. Cheng XT, Li SL, Liu GL, Yang XM, Lu J. Effect of nutritional support on immune function in patients with severe pulmonary infection after renal transplantation. Nan Fang Yi Ke Da Xue Xue Bao 2009; 29:1159-1162.
57. Doley RP, Yadav TD, Wig JD, et al. Enteral nutrition in severe acute pancreatitis. JOP 2009; 10:157-162.
58. Nagata S, Fukuzawa K, Iwashita Y, et al. Comparison of enteral nutrition with combined enteral and parenteral nutrition in post-pancreaticoduodenectomy patients: a pilot study. Nutr J 2009; 8:24.
59. Petrov MS, Loveday BP, Pylypchuk RD, et al. Systematic review and meta-analysis of enteral nutrition formulations in acute pancreatitis. Br J Surg 2009; 96:1243-1252.
60. Ryu J, Nam BH, Jung YS. Clinical outcomes comparing parenteral and nasogastric tube nutrition after laryngeal and pharyngeal cancer surgery. Dysphagia 2009; 24:378-386.
61. Vieira JP, Araújo GF, Azevedo JR, et al. Parenteral nutrition versus enteral nutrition in severe acute pancreatitis. Acta Cir Bras 2010; 25:449-454.
62. Altintas ND, Aydin K, Türkoğlu MA, et al. Effect of enteral versus parenteral nutrition on outcome of medical patients requiring mechanical ventilation. Nutr Clin Pract 2011; 26:322-329.
63. Cangelosi MJ, Auerbach HR, Cohen JT. A clinical and economic evaluation of enteral nutrition. Curr Med Res Opin 2011; 27:413-422.
64. Klek S, Sierzega M, Turczynowski L, et al. Enteral and parenteral nutrition in the conservative treatment of pancreatic fistula: a randomized clinical trial. Gastroenterology 2011;141:157-63, 163.e1.
65. Wang X, Dong Y, Han X, et al. Nutritional support for patients sustaining traumatic brain injury: a systematic review and meta-analysis of prospective studies. PLoS One 2013; 8:e58838.
