# Supplementary material for: Enteral versus parenteral nutrition in critically ill patients: an updated systematic review and meta-analysis of randomized controlled trials
Source: Crit Care. 2016 Apr 29;20:117. doi: 10.1186/s13054-016-1298-1 (PMC4851818; doi:10.1186/s13054-016-1298-1)
Supplement: Additional file 2: Figure A1. — Subgroup analysis comparing the effect of enteral versus parenteral nutrition according to the caloric intake on length of intensive care unit stay (N = 4 studies). Panel A shows the subgroup of aggregated trials in which the caloric intake in the PN group was significantly higher than in the EN group, Panel B shows the subgroup of aggregated trials in which the PN and EN groups received similar caloric intake and Panel C including one trial where caloric intake was not reported. CI confidence interval, EN enteral nutrition, M-H Mantel-Haenszel test, PN parenteral nutrition. Figure A2. Subgroup analysis comparing the effect of enteral versus parenteral nutrition on length of hospital stay according to the caloric intake (N = 7 studies). Panel A shows the subgroup of aggregated trials in which the caloric intake in the PN group was significantly higher than in the EN group, Panel B shows the subgroup of aggregated trials in which the PN and EN groups received similar caloric intake and Panel C including one trial where caloric intake was not reported. CI confidence interval, EN enteral nutrition, IV inverse variance, M-H Mantel-Haenszel test, PN parenteral nutrition. (PDF 148 kb) [file 13054_2016_1298_MOESM2_ESM.pdf]

Enteral versus parenteral nutrition in critically ill patients: an updated systematic review and meta-analysis of randomized controlled trials

Gunnar Elke, Arthur R.H. van Zanten, Margot Lemieux, Michele McCall, Khursheed N. Jeejeebhoy, Matthias Kott, Xuran Jiang, Andrew G. Day, Daren K. Heyland

Additional file 2

Study or Subgroup	EN Mean	EN SD	EN Total	PN Mean	PN SD	PN Total	Weight	Mean Difference IV, Random, 95% CI	Year
**A Caloric intake PN > EN**									
Peterson	3.7	0.8	21	4.6	1	25	68.6%	-0.90 [-1.42, -0.38]	1988
**Subtotal (95% CI)**			**21**			**25**	**68.6%**	**-0.90 [-1.42, -0.38]**	
Heterogeneity: Not applicable									
Test for overall effect: Z = 3.39 (P = 0.0007)									
**B Caloric intake PN ~ EN**									
Adams	13	11	19	10	10	17	0.4%	3.00 [-3.86, 9.86]	1986
Harvey	11.3	12.5	1197	12	13.5	1190	17.0%	-0.70 [-1.74, 0.34]	2014
**Subtotal (95% CI)**			**1216**			**1207**	**17.4%**	**-0.47 [-2.23, 1.29]**	
Heterogeneity: Tau² = 0.58; Chi² = 1.09, df = 1 (P = 0.30); I² = 8%									
Test for overall effect: Z = 0.52 (P = 0.60)									
**C Caloric intake not reported**									
Chen	9.09	2.75	49	9.6	3.06	49	14.0%	-0.51 [-1.66, 0.64]	2011
**Subtotal (95% CI)**			**49**			**49**	**14.0%**	**-0.51 [-1.66, 0.64]**	
Heterogeneity: Not applicable									
Test for overall effect: Z = 0.87 (P = 0.39)									
**Treatment effect, all studies**									
**Total (95% CI)**			**1286**			**1281**	**100.0%**	**-0.80 [-1.23, -0.37]**	
Heterogeneity: Tau² = 0.00; Chi² = 1.60, df = 3 (P = 0.66); I² = 0%									
Test for overall effect: Z = 3.62 (P = 0.0003)									
Test for subgroup differences: Chi² = 0.52, df = 2 (P = 0.77), I² = 0%									

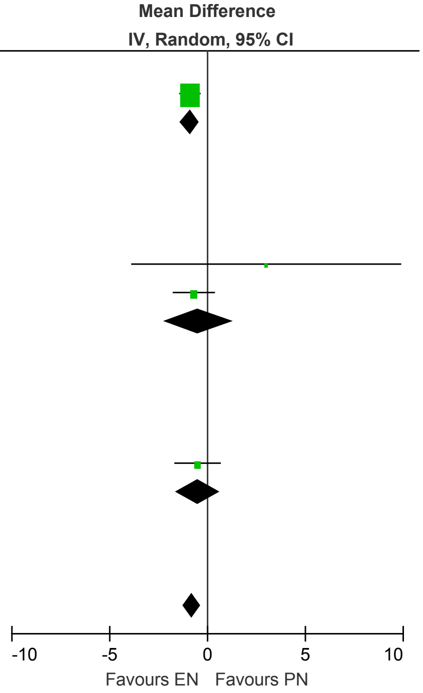

Figure A1. Subgroup analysis comparing the effect of enteral vs. parenteral nutrition according to the caloric intake on length of intensive care unit stay (N = 4 studies). Panel A shows the subgroup of aggregated trials in which the caloric intake in the PN group was significantly higher than in the EN group, Panel B shows the subgroup of aggregated trials in which the PN and EN groups received similar caloric intake and Panel C including one trial where caloric intake was not reported. *CI* confidence interval, *EN* enteral nutrition, *IV* inverse variance, *PN* parenteral nutrition, *SD* standard deviation

Study or Subgroup	EN Mean	EN SD	EN Total	PN Mean	PN SD	PN Total	Weight	Mean Difference IV, Random, 95% CI	Year
**A Caloric intake PN > EN**									
Peterson	13.2	1.6	21	14.6	1.9	21	62.8%	-1.40 [-2.46, -0.34]	1988
Kudsk	20.5	19.9	51	19.6	18.8	45	1.4%	0.90 [-6.85, 8.65]	1992
Woodcock	33.2	43	16	27.3	18.7	18	0.2%	5.90 [-16.87, 28.67]	2001
**Subtotal (95% CI)**			**88**			**84**	**64.4%**	**-1.34 [-2.39, -0.29]**	
Heterogeneity: Tau² = 0.00; Chi² = 0.72, df = 2 (P = 0.70); I² = 0%									
Test for overall effect: Z = 2.50 (P = 0.01)									
**B Caloric intake PN ~ EN**									
Adams	30	21	19	31	29	17	0.3%	-1.00 [-17.71, 15.71]	1986
Borzotta	39	23.1	28	36.9	14	21	0.8%	2.10 [-8.34, 12.54]	1994
Harvey	26.8	33.2	1186	27.5	33.9	1185	11.0%	-0.70 [-3.40, 2.00]	2014
**Subtotal (95% CI)**			**1233**			**1223**	**12.0%**	**-0.54 [-3.12, 2.05]**	
Heterogeneity: Tau² = 0.00; Chi² = 0.26, df = 2 (P = 0.88); I² = 0%									
Test for overall effect: Z = 0.41 (P = 0.68)									
**C Caloric intake not reported**									
Chen	23.32	5.6	49	22.24	3.27	49	23.6%	1.08 [-0.74, 2.90]	2011
**Subtotal (95% CI)**			**49**			**49**	**23.6%**	**1.08 [-0.74, 2.90]**	
Heterogeneity: Not applicable									
Test for overall effect: Z = 1.17 (P = 0.24)									
**Treatment effect, all studies**									
**Total (95% CI)**			**1370**			**1356**	**100.0%**	**-0.67 [-1.57, 0.24]**	
Heterogeneity: Tau² = 0.05; Chi² = 6.12, df = 6 (P = 0.41); I² = 2%									
Test for overall effect: Z = 1.44 (P = 0.15)									
Test for subgroup differences: Chi² = 5.14, df = 2 (P = 0.08), I² = 61.1%									

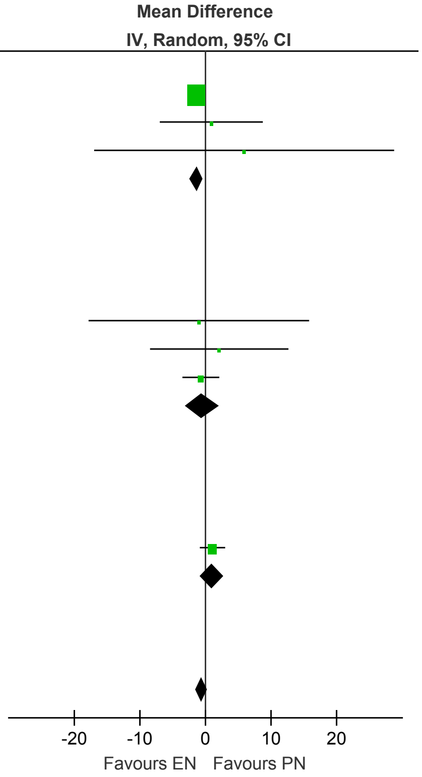

Figure A2. Subgroup analysis comparing the effect of enteral vs. parenteral nutrition according to the caloric intake on length of hospital stay (N = 7 studies). Panel A shows the subgroup of aggregated trials in which the caloric intake in the PN group was significantly higher than in the EN group, Panel B shows the subgroup of aggregated trials in which the PN and EN groups received similar caloric intake and Panel C including one trial where caloric intake was not reported. *CI* confidence interval, *EN* enteral nutrition, *IV* inverse variance, *PN* parenteral nutrition, *SD* standard deviation
