# Supplementary material for: Enteral versus parenteral nutrition in critically ill patients: an updated systematic review and meta-analysis of randomized controlled trials
Source: Crit Care. 2016 Apr 29;20:117. doi: 10.1186/s13054-016-1298-1 (PMC4851818; doi:10.1186/s13054-016-1298-1)
Supplement: Additional file 3: Figure A3. — Subgroup analysis comparing the effect of enteral versus parenteral nutrition on infectious complications in higher versus lower quality trials (with the median methodological score 7 as cutoff). CI confidence interval, EN enteral nutrition, M-H Mantel-Haenszel test, PN parenteral nutrition. (PDF 87 kb) [file 13054_2016_1298_MOESM3_ESM.pdf]

Enteral versus parenteral nutrition in critically ill patients: an updated systematic review and meta-analysis of randomized controlled trials

Gunnar Elke, Arthur R.H. van Zanten, Margot Lemieux, Michele McCall, Khursheed N. Jeejeebhoy, Matthias Kott, Xuran Jiang, Andrew G. Day, Daren K. Heyland

Additional file 3

Study or Subgroup	EN Events	EN Total	PN Events	PN Total	Weight	Risk Ratio M-H, Random, 95% CI	Year
**A Score > 7**							
Adams	15	23	17	23	18.2%	0.88 [0.60, 1.30]	1986
Moore	5	29	11	30	7.4%	0.47 [0.19, 1.19]	1989
Kudsk	9	51	18	45	10.8%	0.44 [0.22, 0.88]	1992
Kalfarentzos	5	18	10	20	8.2%	0.56 [0.23, 1.32]	1997
Woodcock	6	16	11	21	9.8%	0.72 [0.34, 1.52]	2001
Casas	1	11	3	11	1.9%	0.33 [0.04, 2.73]	2007
Harvey	194	1197	194	1191	23.8%	0.99 [0.83, 1.19]	2014
**Subtotal (95% CI)**		**1345**		**1341**	**80.1%**	**0.76 [0.58, 0.99]**	
Total events	235		264				
Heterogeneity: Tau² = 0.04; Chi² = 9.52, df = 6 (P = 0.15); I² = 37%							
Test for overall effect: Z = 2.02 (P = 0.04)							
**B Score ≤ 7**							
Young	5	28	4	23	5.0%	1.03 [0.31, 3.39]	1987
Peterson	2	21	8	25	3.7%	0.30 [0.07, 1.25]	1988
Chen	5	49	18	49	7.6%	0.28 [0.11, 0.69]	2011
Justo Meirelles	2	12	4	10	3.5%	0.42 [0.10, 1.82]	2011
**Subtotal (95% CI)**		**110**		**107**	**19.9%**	**0.42 [0.23, 0.77]**	
Total events	14		34				
Heterogeneity: Tau² = 0.02; Chi² = 3.19, df = 3 (P = 0.36); I² = 6%							
Test for overall effect: Z = 2.80 (P = 0.005)							
**Total (95% CI)**		**1455**		**1448**	**100.0%**	**0.64 [0.48, 0.87]**	
Total events	249		298				
Heterogeneity: Tau² = 0.09; Chi² = 18.71, df = 10 (P = 0.04); I² = 47%							
Test for overall effect: Z = 2.91 (P = 0.004)							
Test for subgroup differences: Chi² = 3.02, df = 1 (P = 0.08), I² = 66.9%							

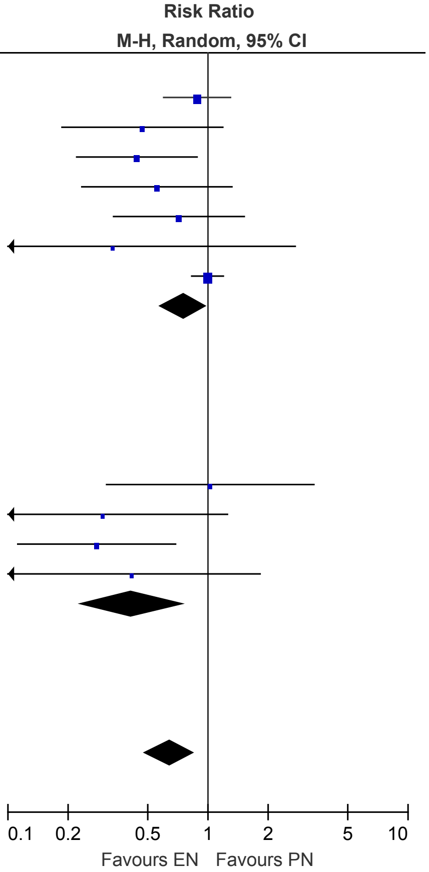

Figure A3. Subgroup analysis comparing the effect of enteral vs. parenteral nutrition on infectious complications in higher vs. lower quality trials (with the median methodological score 7 as cut-off).
CI, confidence interval; EN, enteral nutrition; M-H, Mantel-Haenszel test; PN, parenteral nutrition.
