# Supplementary material for: Enteral versus parenteral nutrition in critically ill patients: an updated systematic review and meta-analysis of randomized controlled trials
Source: Crit Care. 2016 Apr 29;20:117. doi: 10.1186/s13054-016-1298-1 (PMC4851818; doi:10.1186/s13054-016-1298-1)
Supplement: Additional file 4: Figure A4. — Subgroup analysis comparing the effect of enteral versus parenteral nutrition on infectious complications in newer versus older trials (with the publication date 1995 as cutoff). CI confidence interval, EN enteral nutrition, M-H Mantel-Haenszel test, PN parenteral nutrition. (PDF 88 kb) [file 13054_2016_1298_MOESM4_ESM.pdf]

Enteral versus parenteral nutrition in critically ill patients: an updated systematic review and meta-analysis of randomized controlled trials

Gunnar Elke, Arthur R.H. van Zanten, Margot Lemieux, Michele McCall, Khursheed N. Jeejeebhoy, Matthias Kott, Xuran Jiang, Andrew G. Day, Daren K. Heyland

Additional file 4

Study or Subgroup	EN Events	EN Total	PN Events	PN Total	Weight	Risk Ratio M-H, Random, 95% CI	Year
**A Publications > 1995**							
Kalfarentzos	5	18	10	20	8.2%	0.56 [0.23, 1.32]	1997
Woodcock	6	16	11	21	9.8%	0.72 [0.34, 1.52]	2001
Casas	1	11	3	11	1.9%	0.33 [0.04, 2.73]	2007
Justo Meirelles	2	12	4	10	3.5%	0.42 [0.10, 1.82]	2011
Chen	5	49	18	49	7.6%	0.28 [0.11, 0.69]	2011
Harvey	194	1197	194	1191	23.8%	0.99 [0.83, 1.19]	2014
**Subtotal (95% CI)**		**1303**		**1302**	**54.8%**	**0.61 [0.37, 0.99]**	
Total events	213		240				
Heterogeneity: Tau² = 0.17; Chi² = 11.06, df = 5 (P = 0.05); I² = 55%							
Test for overall effect: Z = 1.99 (P = 0.05)							
**B Publications ≤ 1995**							
Adams	15	23	17	23	18.2%	0.88 [0.60, 1.30]	1986
Young	5	28	4	23	5.0%	1.03 [0.31, 3.39]	1987
Peterson	2	21	8	25	3.7%	0.30 [0.07, 1.25]	1988
Moore	5	29	11	30	7.4%	0.47 [0.19, 1.19]	1989
Kudsk	9	51	18	45	10.8%	0.44 [0.22, 0.88]	1992
**Subtotal (95% CI)**		**152**		**146**	**45.2%**	**0.62 [0.39, 0.98]**	
Total events	36		58				
Heterogeneity: Tau² = 0.10; Chi² = 6.57, df = 4 (P = 0.16); I² = 39%							
Test for overall effect: Z = 2.02 (P = 0.04)							
**Total (95% CI)**		**1455**		**1448**	**100.0%**	**0.64 [0.48, 0.87]**	
Total events	249		298				
Heterogeneity: Tau² = 0.09; Chi² = 18.71, df = 10 (P = 0.04); I² = 47%							
Test for overall effect: Z = 2.91 (P = 0.004)							
Test for subgroup differences: Chi² = 0.01, df = 1 (P = 0.94), I² = 0%							

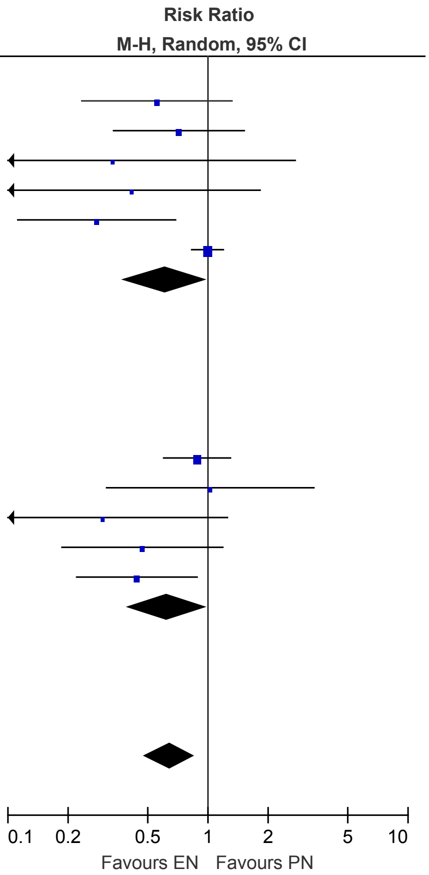

Figure A4. Subgroup analysis comparing the effect of enteral vs. parenteral nutrition on infectious complications in newer vs. older trials (with the publication date 1995 as cut-off).
CI, confidence interval; EN, enteral nutrition; M-H, Mantel-Haenszel test; PN, parenteral nutrition.
